# Seroprevalence of *Coxiella burnetii* in Goats from Central and Western Thailand: Implications for Zoonotic Disease Surveillance and Control

**DOI:** 10.3390/vetsci12121173

**Published:** 2025-12-09

**Authors:** Niorn Ratanapob, Preeda Lertwatcharasarakul, Siriluk Jala, Decha Pangjai, Kridakorn Vongtongsalee, Theera Rukkwamsuk

**Affiliations:** 1 Department of Large Animal and Wildlife Clinical Sciences, Faculty of Veterinary Medicine, Kasetsart University, Kamphaeng Saen Campus, Kamphaeng Saen, Nakhon Pathom 73140, Thailand; 2 Department of Pathology, Faculty of Veterinary Medicine, Kasetsart University, Kamphaeng Saen Campus, Kamphaeng Saen, Nakhon Pathom 73140, Thailand; 3 Kamphaeng Saen Veterinary Diagnostic Center, Faculty of Veterinary Medicine, Kasetsart University, Kamphaeng Saen Campus, Kamphaeng Saen, Nakhon Pathom 73140, Thailand; 4 National Institute of Health, Department of Medical Sciences, Ministry of Public Health, Nonthaburi 11000, Thailand; 5 National Institute of Animal Health, Department of Livestock Development, Ladyao, Chatuchuk, Bangkok 10900, Thailand

**Keywords:** *Coxiella burnetii*, coxiellosis, goats, seroprevalence, Thailand

## Abstract

Goats are important reservoirs of *Coxiella burnetii*, the causative agent of Q fever in humans. Monitoring infection in goats is therefore essential for effective human disease prevention. However, epidemiological data on *C. burnetii* infection in Thai goats remain limited, particularly in the central and western regions, where goat farming has expanded. This study aimed to determine the seroprevalence of *C. burnetii* infection in goats raised in these two regions. A total of 947 serum samples from 101 herds were collected and tested using a commercial enzyme-linked immunosorbent assay (ELISA). Individual- and herd-level seroprevalence were calculated. Pearson’s chi-square test was used to compare infection proportions between regions (central vs. western) and herd types (meat vs. dairy), with *p* < 0.05 considered statistically significant. Overall, individual- and herd-level seroprevalence were 22.39% and 58.42%, respectively. Individual seroprevalence in the central and western regions was 25.16% and 19.71%, while herd-level seroprevalence was 71.15% and 44.90%, respectively. The proportions of infection at both levels were significantly higher in the central region (*p* = 0.044 and *p* = 0.007). In contrast, no significant differences were observed between meat and dairy herds: individual-level seroprevalence was 22.81% and 25.25% (*p* = 0.586), and herd-level seroprevalence was 56.98% and 60.00% (*p* = 0.855), respectively. These findings highlighted the urgent need to strengthen control measures for *C. burnetii* infection in goats in central and western Thailand to protect human, animal, and environmental health.

## Simple Summary

Because coxiellosis vaccines have never been used in Thailand, the antibodies detected in goats indicate natural exposure to *Coxiella burnetii*. This study found a higher individual seroprevalence than previously reported, with increased infection rates observed in the provinces surveyed in earlier studies. This heightened infection risk is likely attributable to expanding goat populations and the use of a more sensitive diagnostic assay. Although within-herd infection levels were lower than those observed during major outbreaks in other countries, the true prevalence may still be underestimated due to the limitations of serological testing. Overall, *C. burnetii* infection appears to be widespread in central and western Thailand, underscoring the urgent need for stronger surveillance, targeted control measures, and increased awareness among at-risk individuals.

## 1. Introduction

Coxiellosis is a multispecies disease caused by *Coxiella burnetii* (*C. burnetii*), a Gram-negative intracellular bacterium [1]. It is a zoonotic infection in which ruminants act as the primary source of human exposure [2]. Human transmission occurs mainly through the inhalation of contaminated aerosols originated from infected birth products or wind-dispersed feces [1]. Although clinical signs are uncommon in ruminants, the disease can lead to substantial economic losses due to reproductive disorders such as late-term abortion, stillbirth, weak offspring, and infertility [1]. The asymptomatic nature of infection makes pathogen elimination within herd particularly challenging [3]. The impact of coxiellosis is generally more severe in goats than in sheep [4]. Both clinically and subclinically infected goats shed large quantities of the pathogen during the periparturient period through birth products, vaginal discharges, feces, urine, and milk [4,5]. Due to its high sensitivity and rapid turnaround time, the enzyme-linked immunosorbent assay (ELISA) is widely used as a screening tool for detecting *C. burnetii* infection in ruminants [6].

*C. burnetii* infection has been reported worldwide. In goats, individual-level seroprevalence ranges from 0.8% to 65.8%, while herd-level seroprevalence ranges from 2.8% to 100% [7,8,9,10,11,12,13,14]. In Thailand, individual-level seroprevalence in goats ranges from 3.5% to 12.8% [15,16,17], and herd-level seroprevalence ranges from 33.3% to 62.0% [16,17,18]. The pathogen has also been detected in sheep, cattle, and buffaloes [15,16,18,19,20]. Among occupationally exposed individuals, seroprevalence has been documented at 12.56% [15]. However, prevalence in these groups is generally lower than that observed in goats. In Thailand, coxiellosis is not currently classified as an immediately notifiable disease. Nonetheless, monitoring and control efforts are conducted within the existing One Health framework involving the Department of Livestock Development (DLD) and the Department of Disease Control (DDC). The DLD enforces general herd biosecurity and sanitation measures such as management of animal waste and isolation of aborting animals, which indirectly mitigate zoonotic risks. The DDC focuses on passive surveillance of acute febrile illness in occupationally exposed individuals.

Goat farming has been increasingly promoted in the central and western regions of Thailand, which together accounted for one-third of the national goat population in 2023 [21]. Due to limited private grazing areas in these regions, some farmers allow their goats to forage on open-access lands. Such practices may facilitate disease transmission, as infected free-roaming goats pose a potential One Health risk to animals, humans, and the environment. Establishing the prevalence of infection in goats was therefore critical to guiding control strategies. However, data from these regions remained scarce. This study aimed to determine the seroprevalence of *C. burnetii* infection in goats raised in central and western Thailand.

## 2. Materials and Methods

An initial sample size of 1000 goats was calculated to provide a reliable estimate of individual seroprevalence, based on an expected prevalence of 12.8% [16] and a 95% confidence level. The calculation was performed using the OpenEpi web-based tool (Version 3.01) and its Sample Size for a Proportion module [22]. This sample size was sufficient to achieve a margin of error of less than 3%. Sample sizes for each province in central and western Thailand were allocated proportionally according to the number of registered meat and dairy goat herds (Table 1). A maximum of 14 samples were collected from each herd. Serum samples were first randomly selected from two laboratories under the DLD. These samples had been collected between 2021 and 2023. All serum samples were placed in serum transport tubes and maintained at 4 °C. Upon arrival, the samples were transferred to −80 °C for long-term storage until subsequent analysis.

Additional blood sampling was conducted in 2023 in the provinces where the number of available samples from DLD laboratories did not meet the target sample size. Whole blood was drawn aseptically from the jugular vein and dispensed into a serum collection tube. The samples were allowed to clot at room temperature before centrifugation. Serum was separated from whole blood by centrifugation using a Thermo Scientific Legend Micro 17 microcentrifuge (Thermo Fisher Scientific, Waltham, MA, USA) at 2000× *g* for 10 min. The serum samples were stored at −80 °C until the diagnostic assay was performed.

A commercial ELISA kit (ID Screen^®^ Q Fever Indirect Multi-species, IDvet™, Grabels, France) was used to detect *C. burnetii*–specific antibodies in serum samples. This diagnostic kit is widely used in animal surveys due to its high throughput and validated performance. All assays were performed according to the manufacturer’s instructions, and positive and negative control serums were included in every run. Optical density (OD) was measured using a Tecan Infinite F50 microplate reader (Tecan Trading AG, Männedorf, Switzerland) at a wavelength of 450 nm.

Samples with a % OD ≥ 51 were considered positive. Samples falling within the range of 40–50% OD were classified as suspect. A herd was classified as positive if at least one sample tested positive. Individual- and herd-level seroprevalence were calculated. Pearson’s chi-square test was used to compare infection proportions between the central and western regions and between meat and dairy goats. A *p* < 0.05 was considered statistically significant.

## 3. Results

A total of 947 goat serum samples from 101 herds in nine central and six western provinces of Thailand were included in the study. The average herd size across the provinces studied was 32.25, ranging from 16.06 to 46.25 (Table 1). Most samples (760 from 83 herds) were obtained from two DLD laboratories, while the remaining 187 samples (18 herds) were collected through additional field sampling. All sampled goats were aged six months or older and included both males and females. However, information regarding reproductive disorders within the sampled herds was not available.

**Figure 1:**
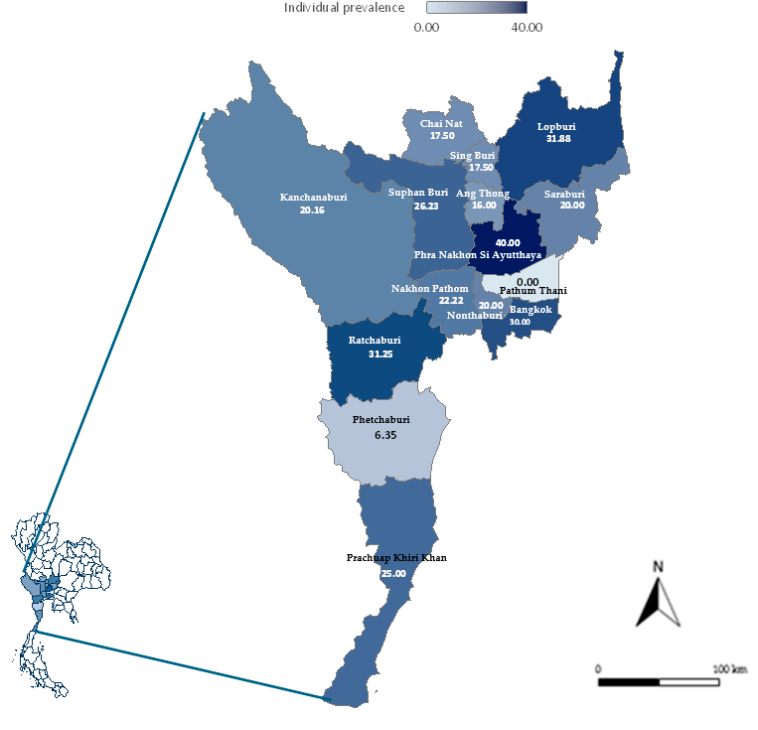
Geographic distribution of individual seroprevalence (%) of *C. burnetii* infection in goats at the provincial level in central and western regions of Thailand.

The overall individual- and herd-level seroprevalence rates of *C. burnetii* infection were 22.39% (95% CI: 19.84–25.16%) and 58.42% (95% CI: 48.43–67.75%), respectively. Twenty-five samples (2.64%) produced suspect results. Within-herd individual seroprevalence ranged from 0% to 100%, with a mean of 22.22%. At the provincial level, the highest individual seroprevalence (40%) was observed in Phra Nakhon Si Ayutthaya, while the highest herd-level seroprevalence (100%) occurred in both Phra Nakhon Si Ayutthaya and Nonthaburi. Pathum Thani, represented by five samples from a single herd, was the only province with no positive samples (Table 1 and Figure 1).

Pearson’s chi-square test showed that both individual- and herd-level seroprevalence rates were significantly higher in the central region compared with the western region (*p* = 0.044 and 0.007, respectively). Although dairy goats exhibited slightly higher individual- and herd-level seroprevalence than meat goats (Table 2), these differences were not statistically significant (*p* = 0.586 and 0.855, respectively).

## 4. Discussion

The final sample count was slightly below the initial target of 1000, primarily because the number of stored serum samples available from the DLD laboratories was lower than anticipated. Nevertheless, total number of samples provided sufficient statistical power, yielding estimates with a margin of error below 3%. Because most samples were obtained from DLD laboratories as part of routine surveillance for other diseases, the sampling process may not have been fully random, introducing potential selection bias. However, the large sample size offers a robust estimate of *C. burnetii* seroprevalence status in goats across central and western Thailand. A key strength of this study was the multi-species applicability of the ID Screen^®^ Q Fever Indirect Multi-species (IDvet™, Grabels, France), which enabled the integration of data into a unified testing database. This consolidated dataset would serve as a foundation for the development of future diagnostic tools, which was an important objective of our research group.

Since coxiellosis vaccines for ruminants have never been used in Thailand, the positive serological results observed in this study reflected natural exposure to *C. burnetii*. The individual seroprevalence of 22.39% reported here was notably higher than the previously reported range of 3.5–12.8% in Thai goats [15,16,17], while the herd-level seroprevalence of 58.42% was comparable to the 62.00% reported in a recent study [16]. Compared with data from 2013 to 2015, our study revealed higher individual and herd-level seroprevalence in the same locations—Kanchanaburi (20.61% and 69.23% vs. 15.66% and 63.16%) and Nakhon Pathom (22.22% and 60.00% vs. 12.10% and 54.54%) [16]. This increase partly attributed to the greater sensitivity of the ELISA used in the present study compared with the previously used immunofluorescent assay [6].

Goat farming promotion programs have resulted in a 2.6-fold increase in the goat population in these provinces between 2015 and 2023 [21]. This rapid expansion likely facilitated pathogen transmission through two primary mechanisms. First, increased animal density may heighten stress and enhance *C. burnetii* dissemination within and between herds. Second, increase animal movement may spread the pathogen more widely, driving up regional prevalence. This high individual seroprevalence observed also suggested active and ongoing transmission within infected herds. The mean individual seroprevalence within infected herds (37.79%) was lower than the 46.6% reported in dairy goat herds in the Netherlands during the major coxiellosis outbreaks from 2007 to 2009 [8]. This difference may reflect variation in the stage of the endemic cycle, with Thailand possibly experiencing a long-standing endemic situation rather than an acute epidemic event. Differences in herd management practices may also contribute to contrasting infection pressures.

Regional differences in seroprevalence may be influenced by herd management practices. A previous study in dairy cattle from two western provinces reported a relatively low individual seroprevalence of 7.33% [23]. Provinces with the highest individual seroprevalence in this study—Lopburi, Ratchaburi, Bangkok, and Suphan Buri—also had large dairy goat populations [21]. However, Pearson’s chi-square test revealed no significant differences in seroprevalence between meat and dairy goats. Although some studies have indicated higher prevalence in dairy ruminants [16,24], the other have reported the opposite trend [14]. The absence of significant differences in our study suggested that goat type alone may not be a strong predictor of infected risk, and management-related factors may play a more influential role. This is important for public health, as it indicates that all goat herds, regardless of type, should be considered high-risk for *C. burnetii* infection, emphasizing the need for comprehensive, sector-wide control measures rather than dairy-focus interventions. A cross-sectional study in the southern Thailand included 105 pregnant women with adverse pregnancy outcomes who provided serum samples before 28 weeks of gestation. Antibodies against *C. burnetii* was detected in 2 (2/105, 1.9%) women, indicating the evidence of potential zoonotic transmission of *C. burnetii* in Thailand [25]. 

Previous studies have identified several factors for *C. burnetii* infection, including increasing age, large herd size, contact with other herds, co-rearing of sheep and goats, tick presence, and poor sanitation of feeding equipment [9,11,14,24,26,27,28,29]. Protective factors include the use of kidding pens, bedding replacement after abortions, and isolation of aborting animals [29]. Infection prevalence has also been shown to vary by breed and geographic region [11,14,24,26,30]. An epidemiological investigation in Northeastern Thailand identified prolonged herd establishment (>5 years) and reproductive disorders as herd-level risk factors; and female sex, crossbred genotype, and anemia as individual-level risk factors [17]. Due to limited background information from DLD-derived samples, risk factor analysis could not be performed in this study. Future research should integrate detailed herd data to better identify risk and protective factors and inform targeted control strategies. Enhanced surveillance of other animal species and humans in affected area is recommended to better understand the epidemiology of coxiellosis. Given the zoonotic nature of *C. burnetii*, the high seroprevalence observed in goats suggested a substantial risk of environmental contamination and human exposure.

Potential limitations of this study included the underestimate of prevalence due to seronegative infected animals, as reported by Rouset et al. [5]. Although the study provided valuable insights into the seroprevalence of *C. burnetii* infection in goats, several factors should be considered when interpreting the findings. First, the investigation relied solely on serological testing and did not incorporate molecular assays such as PCR, which would help confirm the presence of the organism and identify actively shedding animals. Second, the lack of clinical and reproductive outcome data—such as histories of abortion, stillbirths, weak neonates, or other herd health indicators—limited the ability to evaluate the relationship between *C. burnetii* exposure and disease expression at either the individual or herd level. These limitations highlight the need for future studies that integrate serology with molecular detection and detailed herd-level health records to clarify the epidemiological role of infected goats, identify active transmitters, and more accurately assess the public health and economic consequences of coxiellosis in Thailand.

## 5. Conclusions

This study revealed a substantial prevalence of *C. burnetii* infection among both meat and dairy goats in central and western Thailand. These findings highlight the need for strengthened surveillance and effective control measures to reduce pathogen transmission within goat populations. Additionally, raising awareness among at-risk individuals about appropriate personal protective practices is critically important for minimizing exposure and improving overall One Health outcomes.

## Figures and Tables

**Table 1:** Seroprevalence rate of *C. burnetii* infection in goats at individual and herd levels by province.

Province	Total No. of Registered Herds	Mean of Herd Size	No. of Individual Samples	No. of Positive Individuals	Individual Seroprevalence	No. of Herds	No. of Positive Herds	Herd Seroprevalence
Central region	6749	27.74	465	117	25.16	52	37	71.15
Bangkok	545	22.61	50	15	30.00	5	4	80.00
Chai Nat	1135	36.26	80	14	17.50	10	7	70.00
Nonthaburi	283	16.05	20	4	20.00	2	2	100.00
Pathum Thani	126	27.06	5	0	0.00	1	0	0.00
Lopburi	2414	32.24	160	51	31.88	16	12	75.00
Saraburi	871	33.09	60	12	20.00	7	6	85.71
Sing Buri	559	33.12	40	7	17.50	4	1	25.00
Phra Nakhon Si Ayutthaya	401	21.54	25	10	40.00	3	3	100.00
Ang Thong	415	27.72	25	4	16.00	4	2	50.00
Western region	7996	39.02	482	95	19.71	49	22	44.90
Kanchanaburi	3223	38.82	129	26	20.16	13	9	69.23
Nakhon Pathom	324	43.34	54	12	22.22	5	3	60.00
Prachuap Khiri Khan	1152	41.13	20	5	25.00	2	1	50.00
Phetchaburi	853	46.25	126	8	6.35	13	1	7.69
Ratchaburi	852	30.18	80	25	31.25	9	4	44.44
Suphan Buri	1592	34.39	73	19	26.03	7	4	57.14
Overall	14,745	32.25	947	212	22.39	101	59	58.42

**Table 2:** Seroprevalence rate of *C. burnetii* infection in goats at individual and herd levels by production type.

Type	No. of Individual Samples	No. of Positive Individuals	Individual Seroprevalence	No. of Herds	No. of Positive Herds	Herd Seroprevalence
Meat	798	182	22.81	86	49	56.98
Dairy	99	25	25.25	10	6	60.00
Unknown	50	5	10.00	5	4	80.00
Overall	947	212	22.39	101	59	58.42

## Data Availability

The original contributions presented in this study are included in the article. Further inquiries can be directed to the corresponding author.
